# Diversity, enumeration, and isolation of *Arcobacter* spp. in the giant abalone, *Haliotis gigantea*

**DOI:** 10.1002/mbo3.890

**Published:** 2019-06-05

**Authors:** Yukino Mizutani, Shunpei Iehata, Tetsushi Mori, Ryota Oh, Satoshi Fukuzaki, Reiji Tanaka

**Affiliations:** ^1^ Graduate School of Bioresources, Laboratory of Marine Microbiology Mie University Tsu Japan; ^2^ School of Fisheries and Aquaculture Science Universiti Malaysia Terengganu Kuala Terengganu Terengganu Malaysia; ^3^ Department of Biotechnology and Life Science Tokyo University of Agriculture and Technology Koganei Japan

**Keywords:** abalone, *Arcobacter*, clone libraries, cultivation, fluorescent in situ hybridization

## Abstract

*Arcobacter* have been frequently detected in and isolated from bivalves, but there is very little information on the genus *Arcobacter* in the abalone, an important fishery resource. This study aimed to investigate the genetic diversity and abundance of bacteria from the genus *Arcobacter* in the Japanese giant abalone, *Haliotis gigantea*, using molecular methods such as *Arcobacter*‐specific clone libraries and fluorescence in situ hybridization (FISH). Furthermore, we attempted to isolate the *Arcobacter* species detected. Twelve genotypes of clones were obtained from *Arcobacter*‐specific clone libraries. These sequences are not classified with any other known *Arcobacter* species including pathogenic *Arcobacter* spp., *A. butzleri, A. skirrowii,* and *A*. *cryaerophilus*, commonly isolated or detected from bivalves. From the FISH analysis, we observed that ARC94F‐positive cells, presumed to be *Arcobacter,* accounted for 6.96 ± 0.72% of all EUB338‐positive cells. In the culture method, three genotypes of *Arcobacter* were isolated from abalones. One genotype had a similarity of 99.2%–100.0% to the 16S rRNA gene of *Arcobacter marinus*, while the others showed only 93.3%–94.3% similarity to other *Arcobacter* species. These data indicate that abalones carry *Arcobacter* as a common bacterial genus which includes uncultured species.

## Graphical abstract

Diversity of marine *Arcobacter* associated with the giant abalone *Haliotis gigantea*

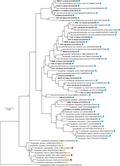

## 1 INTRODUCTION

*Arcobacter*, formerly classified as *Campylobacter*, is a member of the class Epsilonproteobacteria, as proposed by Vandamme et al. (1991). Some *Arcobacter* bacteria have shown pathogenicity to humans, and thus many studies have focused on livestock. Species isolated from pork, broiler carcasses, cattle, ducks, human stool, or porcine abortions include: *Arcobacter butzleri*, *A. skirrowii, A. cibarius, A. cryaerophilus*, *A*. *trophiarum*, *A*. *defluvii*, *A*. *thereius*, *A*. *suis*, *A*. *cloacae*, *A*. *lanthieri*, *A*. *faecis*, *A*. *lacus,* and *A*. *caeni* (Collado, Levican, Perez, & Figueras, 2011; De Smet et al., 2011; Houf et al., 2005, 2009; Kiehlbauch et al., 1991; Levican, Collado, & Figueras, 2013; Neill, Campbell, O'Brien, Weatherup, & Ellis, 1985; Pérez‐Cataluña, Salas‐Massó, & Figueras, 2018b; Vandamme et al., 1992; Whiteduck‐Léveillée et al., 2015, 2016). Among them, *A*. *butzleri*, *A*. *cryaerophilus,* and *A*. *skirrowii* are considered to be of clinical interest because they are associated with gastrointestinal disease and bacteremia in humans, and with reproduction disorders, mastitis, and gastric ulcers in farm animals (Ho, Lipman, & Gaastra, 2006). *Arcobacter thereius* was also isolated from porcine abortions, but the pathological potential of this species is still unknown (Houf et al., 2009). In contrast, other species have not been directly associated with animal or human diseases.

Recently, *Arcobacter* spp. have also been isolated from marine environments, such as seawater and coastal sediments, and from marine invertebrates. To date, 18 *Arcobacter* species from a total of 29 have been isolated from marine environments (Table 1), suggesting that this environment may be one of the main habitats for this genus. *Arcobacter* are found in bivalves (Collado, Guarro, & Figueras, 2009; Laishram, Rathlavath, Lekshmi, Kumar, & Nayak, 2016; Levican, Collado, Yustes, Aguilar, & Figueras, 2014; Salas‐Massó, Andree, Furones, & Figueras, 2016). Romero, García‐Varela, Laclette, and Espejo (2002) reported *Arcobacter* spp. are widespread in the Chilean oyster in their analysis using 16S rRNA‐RFLP. In addition to bivalves, it has been reported that *Arcobacter* spp. are found in European lobsters and abalone (Meziti, Mente, & Kormas, 2012; Tanaka, Ootsubo, Sawabe, Ezura, & Tajima, 2004). These results suggest that *Arcobacter* spp. are widely distributed in marine invertebrates, and potentially indigenous bacteria may play some important role in the host. However, knowledge on the presence and diversity of *Arcobacter* associated with marine invertebrates including abalone is still lacking compared to pathogenic *Arcobacter*.

**Table 1:** List of *Arcobacter* spp. isolated from different marine environments

Source	Species	Reference
Roots of *Spartina alterniflora*, sediments from salt marshes	*A. nitrofigilis* CI	McClung, Patriquin, and Davis (1983)
Water from hypersaline lagoon	*A. halophilus* LA31B	Donachie, Bowman, On, and Alam (2005)
Mussels, brackish water	*A. mytili* F2075	Collado, Cleenwerck, Trappen, Vos, and Figueras (2009)
Seawater, seaweeds, and starfish	*A. marinus* CL‐S1	Kim et al. (2010)
Sewage	*A. defluvii* SW28‐11	Collado et al. (2011)
Mussels	*A. ellisii* F79‐6	Figueras, Levican, Collado, Inza, and Yustes (2011)
Mussels and oysters	*A. molluscorum* F98‐3	Figueras, Collado, et al. (2011)
Clams	*A. venerupis* F67‐11	Levican et al. (2012)
Mussels	*A. bivalviorum* F4	Levican et al. (2012)
Mussels and sewage	*A. cloacae* SW28‐13	Levican et al. (2013)
Estuarine sediment	*A. anaerophilus* JC84	Sasi Jyothsna, Rahul, Ramaprasad, Sasikala, and Ramana (2013)
Mussels	*A. ebronensis* F128‐2	Levican, Rubio‐Arcos, Martinez‐Murcia, Collado, and Figueras (2015)
Seawater	*A. aquimarinus* W63	Levican et al. (2015)
Seawater	*A. acticola* AR‐13	Park, Jung, Kim, and Yoon (2016)
Seawater	*A. pacificus* SW028	Zhang, Yu, Wang, Yu, and Zhang (2016)
Great scallop larvae and tank seawater	*A. lekithochrous* LFT 1.7	Diéguez et al. (2017)
Abalone	*A. lekithochrous* MA5 (syn. *A. haliotis* MA5)	Tanaka et al. (2017)
Sewage	*A. canalis* F138‐33	Pérez‐Cataluña, Salas‐Masso, and Figueras (2018a)

Therefore, in order to gain knowledge on *Arcobacter* spp. in marine invertebrates, we tried to explore the diversity and abundance of the genus *Arcobacter* in the giant abalone *Haliotis gigantea*, an important fishery resource inhabiting shallow water environments. We used cultivation‐independent methods, such as *Arcobacter*‐specific clone libraries and fluorescence in situ hybridization (FISH). We also attempted to isolate *Arcobacter* strains using selective cultivation, and report here the genetic relationships between successfully isolated strains.

## 2 MATERIALS AND METHODS

### 2.1 Sample collection and DNA extraction

Nine cultivated giant abalones, *H. gigantea*, (sample code: CA) and rearing water samples from two tanks (sample code: RW) were collected from the Owase Farming Fishery Center (Owase, Mie, Japan) in February 2012. These samples were supplied for construction of *Arcobacter*‐specific clone libraries and for FISH. For isolation of *Arcobacter* spp., three endemic *H. gigantea* specimens were collected from fish markets in Mie, Japan, in September 2017.

The internal organs, including the gut and gills, were collected from the abalones followed by the previously described method (Tanaka et al., 2004). To tear off bacterial cells from their host tissue, we used a beads beater on the condition of slower stroke and shorter time (We state that the method will not allow obtaining the whole bacterial diversity present in the host tissue as compared to an approach based on completely grinding the host tissue). Abalone specimens were pooled into each tube and homogenized using a beads beater (4,200 rpm, 30 s; Tietech Co., Nagoya, Japan). Host tissues were removed from CA samples by quick centrifugation (1 s, 8,000 *g*), and the supernatant was transferred to new tubes and centrifuged for 20 min at 15,000 *g* to recover bacterial cells. Rearing water samples (RW) were concentrated (50×) using 0.22 µm cellulose membrane filters (Advantec, Tokyo, Japan) and resuspended in sterile phosphate‐buffered saline (PBS: 130 mM NaCl, 10 mM Na_2_HPO_4_/NaH_2_PO_4_; pH 7.4). Aliquots of bacterial pellets thus obtained from CA and RW samples were subsequently used for FISH analysis and DNA extraction. Bacterial genomic DNA from each sample was extracted using Promega DNA purification system (Promega Corp., Madison, WI, USA) according to the manufacturer's instructions.

### 2.2 PCR analysis, construction of *Arcobacter*‐specific clone libraries and sequencing

The *Arcobacter* genus‐specific primers, ARC94F primer (5′‐TGCGCCACTTAGCTGACA‐3′) and ARC1446R primer (5′‐TAGCATCCCCGCTTCGAATGA‐3′) (Harmon & Wesley, 1996; Snaidr, Amann, Huber, Ludwig, & Schleifer, 1997) were used to amplify the *Arcobacter* 16S rRNA gene from each sample. PCR reaction mixtures contained 1× PCR reaction buffer, 200 µM dNTP, 5 pmol of each primer, 2.5 units Ex Taq polymerase (TaKaRa Biotechnology Corp., Kyoto, Japan), and 10–100 ng of DNA for a total volume of 50 µl. PCR reactions were performed using an iCycler (Bio‐Rad Lab., Hercules, CA, USA). The amplification conditions were as follows: initial denaturation of 4 min at 95°C followed by 25 cycles of denaturation for 30 s at 95°C, primer annealing for 30 s at 55°C, and primer extension at 72°C for 1.5 min. This was followed by a final extension reaction at 72°C for 7 min. Distilled H_2_O was used as the template for negative controls; these produced no PCR product, indicating the absence of contaminating DNA in reactions and reagents. The 16S rRNA gene amplicons were purified by the PCR Preps DNA Purification System (Promega Corp.) according to the manufacturer's instructions, and subsequently ligated into the TOPO TA cloning vector (Invitrogen Corp., Carlsbad, CA, USA). Ligation products were transformed into *Escherichia coli* One Shot TOP10 cells (Invitrogen Corp.) and screened for plasmid insertions by following the manufacturer's instructions. Plasmid DNA containing insertions was sequenced with the ARC94F primer using the Sanger method with an ABI 3730 sequencer (Applied Biosystems, Foster City, CA, USA). Chromatograms of DNA sequences were examined using Chromas v2.3.3 (Technelysium Pty Ltd., South Brisbane, Australia). All sequences were examined for chimerism using a chimeric sequences detection tool, Bellerophon (Huber, Faulkner, & Hugenholtz, 2004).

### 2.3 Fluorescent in situ hybridization

The total number of *Arcobacter* cells in abalone samples was counted using the FISH method (Kepner & Pratt, 1994). Aliquots of bacterial pellets obtained from CA and RW samples were rinsed by 600 µl of sterile PBS and centrifuged at 12,000 *g* for 5 min, then removed supernatant. These procedures were performed two times. Samples were fixed by adding one volume of PBS to three volumes of 4% paraformaldehyde/PBS, and incubating at 4°C for 3 hr. After centrifugation, the fixative was removed and the bacterial pellet was washed twice with PBS. The washed cells were mixed with 1× PBS and 96% EtOH (1:1) and stored at −20°C (Roller, Wagner, Amann, Ludwig, & Schleifer, 1994). Three microliters of fixed‐cell suspension was spread on the well of an aminopropyl‐silane‐coated 8‐well slide (Matsunami, Japan). The slides were air‐dried and dehydrated by successive immersion in 50, 80, and 99.5%. Hybridization was performed based on a previous study by Ootsubo et al. (2003), with modifications. A 5′‐end TAMRA‐labeled ARC94F probe (5′‐TGCGCCACTTAGCTGACA‐3′; Sigma‐Aldrich Corp, St. Louis, MO; Moreno et al., 2003) was designed to target the 16S rRNA of *Arcobacter* spp., and a 5′‐end FITC‐labeled EUB338 probe (5′‐GCTGCCTCCCGTAGGAGT‐3′; Sigma‐Aldrich Corp; Amann et al., 1990) to target the 16S rRNA gene of most members of the domain Bacteria. Prior to hybridization, each probe was added to pre‐warmed hybridization buffer (0.9 M NaCl, 20% formamide, 20 mM Tris–HCl [pH 7.4] and 0.1% sodium dodecyl sulfate [SDS]) to a final concentration of 10 p.m. The probe solution was spread on each hybridization well, and the slides incubated at 46°C for 3 hr in an MHS‐2000 hybridization oven (EYELA, Tokyo, Japan). After hybridization, each well was washed twice with washing buffer (20 mM Tris‐HCl [pH 7.4], 180 mM NaCl and 0.01% SDS), rinsed with ddH_2_O and air‐dried. An epifluorescence light microscope (Eclipse 400; Nikon Corp., Tokyo, Japan), was used for observing the stained cells. Due to a technical error during sampling, we were unable to detect the mean ± SE from RW samples.

### 2.4 Isolation of *Arcobacter* spp.

For the isolation of *Arcobacter* species, the procedure described by Salas‐Massó et al. (2016) was followed, but the media was slightly modified by changing 2.5% NaCl to artificial seawater. The bacterial mixture from three abalones was diluted 10 times using sterile Daigo's Artificial Seawater SP (Nihon Pharmaceutical Co., Tokyo, Japan) and 100 µl of the diluted mixture was inoculated into *Arcobacter* Broth (Oxoid Ltd., Hampshire, UK, USA) with CAT supplement [cefoperazone at 8 mg/L, amphotericin B at 10 mg/L and teicoplanin at 4 mg/L] (Oxoid Ltd., Atabay & Corry, 1997) suspended in 75% Daigo's Artificial Seawater SP instead of distilled water with 2.5% NaCl. The culture solutions were incubated for 48 or 96 hr at 15°C (sample codes: 15T48H and 15T96H, respectively) or 25°C (sample codes: 25T48H and 25T96H) under aerobic conditions. After cultivation, 200 µl of post‐cultured broth was pipetted onto the surface of polycarbonate membrane filters (pore size, 0.4 µm: Merck Millipore, Burlington, MA) placed on Marine Agar 2216 (Difco, Detroit, MI, USA). The plates were incubated at room temperature for 30 min to allow passive filtration (Atabay & Corry, 1997). Next, the filters were carefully removed and the flow‐through was spread on Marine Agar 2216. The media was incubated at the same temperature and time as the primary culture. After cultivation, presumed *Arcobacter* colonies (tiny and beige to off‐white in color) were selected and applied to colony PCR in the same way as *Arcobacter*‐specific clone libraries using ARC94F and ARC1446R primers. Positive PCR products were sequenced using standard Sanger sequencing.

### 2.5 Cluster analysis of the bacterial community structure

For each sample, sequences were aligned and grouped in Operational Taxonomic Units (OTUs) with >97% sequence identity (Stackebrandt & Goebel, 1994). Homology searches were performed using sequences of approximately 700 bp and the highest homology sequences with each OTU were chosen as the closest relatives. All BLASTn searches were performed with the default parameters available through the National Center for Biotechnology Information website (http://www.ncbi.nlm.nih.gov/). Multiple alignments and calculation of distant matrixes were performed by CLUSTAL W (Thompson, Higgins, & Gibson, 1994), using MEGA 7.0 (Kumar, Stecher, & Tamura, 2016). A phylogenetic tree was constructed using the maximum‐likelihood method of MEGA 7.0, with 1,000 replicates in the bootstrap analysis and Kimura's two‐parameter model (Kimura, 1980). Distances were estimated with the Jukes‐Cantor correction.

## 3 RESULTS

### 3.1 *Arcobacter*‐specific clone libraries

To perform a comprehensive search for *Arcobacter* spp. from abalone and their surrounding seawater, we established *Arcobacter*‐specific 16S rRNA gene clone libraries. A total of 120 and 30 clones were obtained in our study from abalone (CA) and rearing water (RW) samples, respectively (Table 2). Among these clones, *Arcobacter* sequences were observed as 12 OTUs from CA and seven OTUs from RW.

**Table 2:** 16S rRNA gene sequences identified in the clone library and isolation from abalone or seawater

Methods	Samples	OTUs	No. of clones or isolates	Highest similarity sequence (accession number)	Identity (%)
*Arcobacter*‐specific clone libraries	Abalone (CA)	CA1	16	Uncultured bacterium clone KSTye‐VF1‐B‐003 (JQ611206)	100
		CA2	3	*Arcobacter* sp. EP1 (LT629996)	98.6
		CA3	17	Uncultured bacterium clone SF‐July‐156 (HM591463)	98.8
		CA4	3	Uncultured *Arcobacter* sp. clone DVASD_D318 (KF463610)	98.9
		CA5	3	Uncultured *Arcobacter* sp. clone DVBSW_D345 (KF722009)	99.5
		CA6	3	Uncultured bacterium clone AJ‐U‐CD‐41(H) (JX170315)	98.4
		CA7	20	Uncultured bacterium clone AJ‐U‐CD‐41(H) (JX170315)	100
		CA8	1	Uncultured bacterium clone TopBa31 (EF999357)	97.2
		CA9	24	Uncultured epsilon‐proteobacterium clone AT‐pp13 (AY225610)	95.2
		CA10	4	Uncultured epsilon‐proteobacterium clone PI_4z10e (AY580424)	99.6
		CA11	25	Uncultured bacterium clone SF‐July‐74 (HM591442)	99.2
		CA12	1	Epsilon‐proteobacterium Yb‐*F* (AB496655)	100
	Rearing water (RW)	RW1	12	Uncultured bacterium clone HF071 (JX391310)	98.7
		RW4	4	Uncultured epsilon‐proteobacterium clone PI_4z7d (AY580420)	98.3
		RW5	4	*Arcobacter pacificus* SW028 (JN118552)	98.5
		RW6	3	Uncultured bacterium clone SF‐July‐156 (HM591463)	97.9
		RW10	4	*Arcobacter bivalviorum* F4 (FJ573217)	97.4
		RW17	2	Uncultured bacterium clone C13W_197 (HM057704)	98.8
		RW20	1	Uncultured marine bacterium clone B‐Alg40 (HM437504)	99.8
Isolations	Abalone 15°C (15T96H)	15T96H‐1	4	Uncultured bacterium clone HglApr921 (JX016315)	98.2
		15T96H‐2	1	Uncultured bacterium clone HglApr921 (JX016315)	98.3
	Abalone 25°C (25T96H)	25T96H	5	*Arcobacter marinus* strain CL‐S1 (EU512920)	100

The 16S rRNA genes identified from abalone (CA) using the *Arcobacter*‐specific clone library did not show high similarity to known species within the NCBI database. The most abundant genotype in CA was CA11 (25 clones, accession number: LC133145), which had a high similarity score of 99.2% to uncultured bacterium clone SF‐July‐74 (HM591442). CA1 (LC133157) had 100% similarity to bacterium clone KSTye‐VF1‐B‐003 (JQ611206) collected from venting fluid in a yellow vent off Kueishan Island, while CA3 and CA12 (LC133141 and LC133148) each had a similarity of 98.8% and 100% to uncultured bacterium clone SF‐July‐156 (HM591463) and epsilon‐proteobacterium Yb‐F (AB496655) collected from seawater. CA3 did not cluster with any sequences. CA4, CA5, CA8, and CA9 (LC133159, LC133146, LC133160, and LC133142) were assigned to uncultured bacteria collected from marine environments: CA4 and CA5 showed similarity of 98.9% and 99.5% to uncultured *Arcobacter* sp. clones DVASD_D318 and DVBSW_D345, respectively (KF463610 and KF722009) from a marine coastal ecosystem, CA8 showed 97.2% similarity to uncultured bacterium clone TopBa31 (EF999357) from Pearl River Estuary sediments, and CA9 showed 95.2% similarity to uncultured epsilon‐proteobacterium clone AT‐pp13 (AY225610) from pumice fragments exposed to a Mid‐Atlantic Ridge vent. CA6 and CA7 (LC133158 and LC133140) were assigned to uncultured bacterium clone AJ‐U‐CD‐41(H) (JX170315) isolated from the intestine of a sea cucumber, *Apostichopus japonicas*, with 98.4% and 100% similarity, respectively. Finally, CA2 and CA10 (LC133161 and LC133156) were closely affiliated with an isolate or a clone collected from protists. CA2 had 98.6% similarity to *Arcobacter* sp. EP1 (LT629996) isolated from the epibiont of unicellular protists, and CA10 had 99.6% similarity to uncultured epsilon‐proteobacterium clone PI_4z10e from coastal bacterioplankton sampled at Plum Island Sound Estuary.

In the rearing water samples, RW5 (LC133151) showed 98.5% sequence similarity to *A*. *pacificus* strain SW028 isolated from seawater (JN118552), and RW10 (LC133153) had a 97.4% high homology to *A*. *bivalviorum* strain F4 isolated from bivalves (FJ573217). RW1 (LC133149) showed a sequence similarity of 98.7% to uncultured bacterium clone HF071 (JX391310) detected from marine sediment. RW4 (LC133150) was affiliated with uncultured epsilon‐proteobacterium clone PI_4z7d in a coastal bacterioplankton sample from Plum Island Sound Estuary, at 98.3% similarity. RW17 (LC133154) was closely related to uncultured bacterium clone C13W_197 (HM057704) collected from seawater, at 98.8% similarity. RW6 (LC133152) showed 97.9% similarity to uncultured bacterium clone SF‐July‐156 (HM591463), and RW20 (LC133155) was closely related at 99.8% similarity to uncultured marine bacterium clone B‐Alg40 (HM437504) detected from the surface of algae.

### 3.2 Detection of ARC94F‐positive cells by FISH

**Figure 1:**
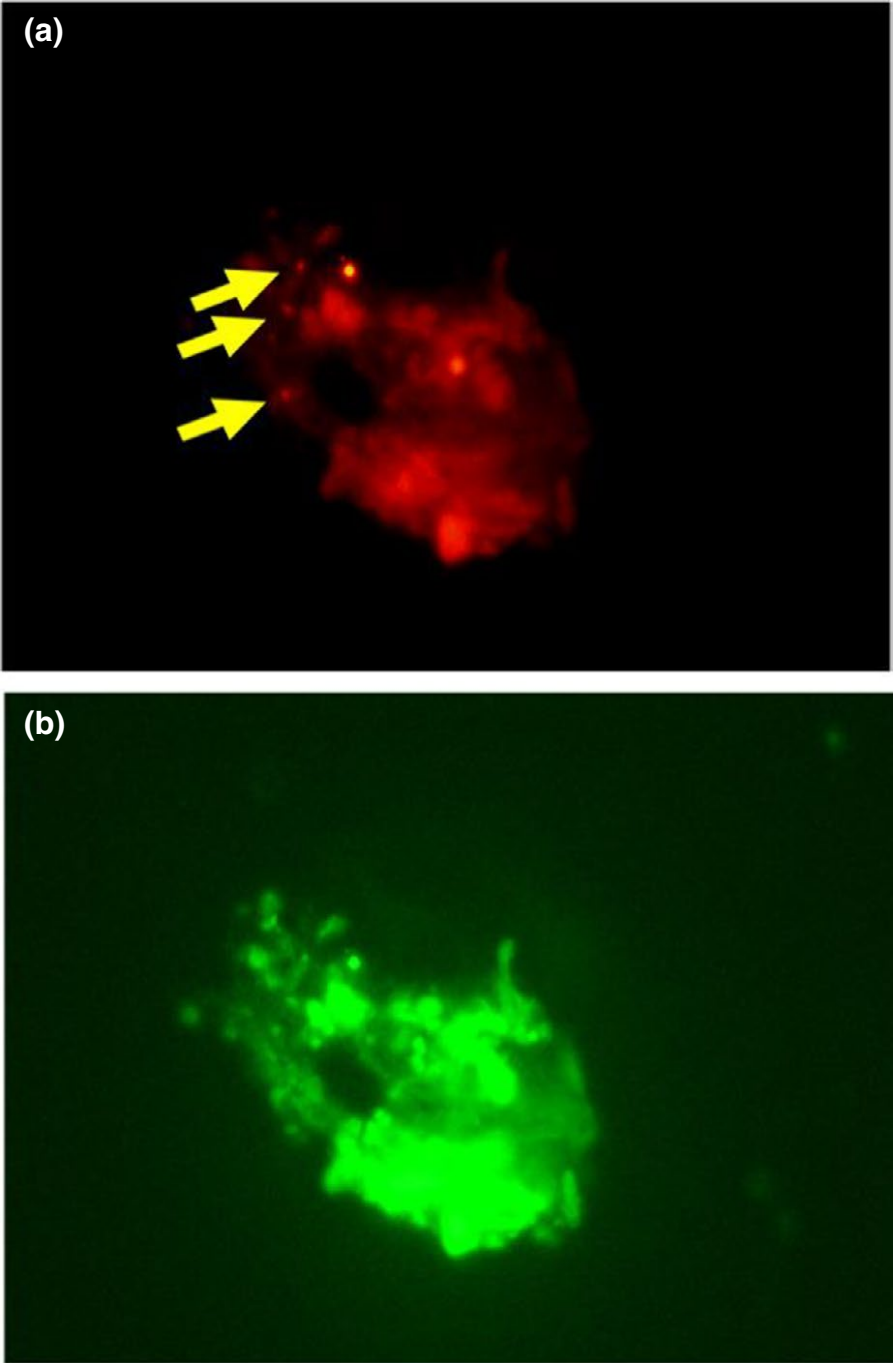
FISH photograph using (a) probe ARC94F and (b) probe EUB338, showing positive cells attached to abalone tissues. Yellow arrows indicate *Arcobacter*‐stained cells. FISH, fluorescence in situ hybridization

The amount of *Arcobacter* spp. in homogenized cultured abalone and rearing water samples from Minami‐ise, Mie, Japan, was determined by FISH (Table 3). From the abalone samples (*n* = 9), with a total bacteria count of 1.18 ± 0.71 × 10^7^ cells/g, the number of ARC94F‐positive bacteria was 8.06 ± 0.05 × 10^5^ cells/g, dominating the total bacteria count at 6.96 ± 0.72% (Figure 1). In the abalone rearing water (*n* = 2), the total bacteria count for each sample was 2.76 × 10^4^ cells/ml and 4.63 × 10^4^ cells/ml, respectively. Among these, the number of ARC94F‐positive bacteria was 1.33 × 10^3^ cells/ml and 1.08 × 10^3^ cells/ml, dominating 4.8% or 2.3% of the total bacteria, respectively.

**Table 3:** Total and *Arcobacter* bacterial counts from abalones or in rearing water by direct microscopy

	Abalone (*n* = 9, mean ± SE)	Rearing water (*n* = 2, mean)
EUB338 (cells/g or ml)	1.18 ± 0.71 × 10^7^	3.70 × 10^4^
ARC94 (cells/g or ml)	8.06 ± 0.05 × 10^5^	1.21 × 10^3^
Rate of *Arcobacter* (% of total bacterial count)	6.96 ± 0.72	3.55

### 3.3 Isolation of *Arcobacter* Spp.

When incubation of samples from natural abalones collected in Mie, Japan, was performed for 96 hr at 15°C or 25°C (sample codes: 15T96H or 25T96H), the total numbers of colonies were 871 and 338, respectively. Upon further selection, 12 and eight colonies from the 15T96H and 25T96H samples were presumed to be *Arcobacter* colonies (tiny and beige to off‐white in color). Colony PCR subsequently confirmed that five of the selected *Arcobacter*‐like colonies for each sample were affiliated to *Arcobacter* spp. Finally, colonies with sequence similarity of >97% were grouped and given a similar designation. We obtained four strains designated as 15T96H‐1, one strain designated as 15T96H‐2 from the 15T96H sample, and five strains designated as 25T96H‐1 from the 25T96H sample. The 16S rRNA gene sequences of 15T96H‐1 and 15T96H‐2 showed high similarity (98.2% and 98.3%, respectively) with uncultured bacterium clone HglApr921 (JX016315). Since both these strains have low homology with other isolated *Arcobacter* species (93.3%–94.3% similarity), they suggest potential new species. On the other hand, all strains of 25T96H‐1 showed similarity ranging from 99.2% to 100% with *A. marinus*. For the samples incubated at 15°C or 25°C for 48 hr, a few colonies were isolated but they were not identified as *Arcobacter* spp.

## 4 DISCUSSION

Thus far, research on *Arcobacter* has mainly been focused on detection since several *Arcobacter* species are pathogenic to humans (Collado et al., 2011; Ho et al., 2006; Houf, Tutenel, Zutter, Hoof, & Vandamme, 2000). Meat and seafood are the most common sources of *Arcobacter* reported (Atabay & Corry, 1997; Houf, Zutter, Hoof, & Vandamme, 2002; Hume et al., 2001; Romero et al., 2002; Collado, Guarro, et al., 2009; Levican et al., 2014; Salas‐Massó et al., 2016; Mottola et al., 2016; Rathlavath, Kohli, et al., 2017; Rathlavath, Kumar, & Nayak, 2017; Vicente‐Martins, Oleastro, Domingues, & Ferreira, 2018). *Arcobacter* is also detected or isolated from seawater, sewage, and drinking water, and these environments are considered important as they could be one of the possible routes of transmission of *Arcobacter* to human and animal intestinal tracts (Collado et al., 2011). Various methods including enterobacterial repetitive intergenic consensus PCR (ERIC‐PCR), randomly amplified polymorphic DNA‐PCR (Houf et al., 2002), and amplified fragment length polymorphism (On, Harrington, & Atabay, 2003,2004) have been used to detect and elucidate the transmission routes or to trace the sources of *Arcobacter* outbreaks (Collado & Figueras, 2011). Multiplex‐PCR that can detect multiple species simultaneously has also been used (Brightwell et al., 2007; Houf et al., 2000; Khan et al., 2017). ERIC‐PCR in particular has been successfully applied to outbreak investigations (Vandamme et al., 1993) in food. Although these methods have advantages due to its simplicity and cost, they detect only a specific species from isolated strains or from mixed cultures based on specific culture conditions (González, Bayas Morejón, & Ferrús, 2017).

**Figure 2:**
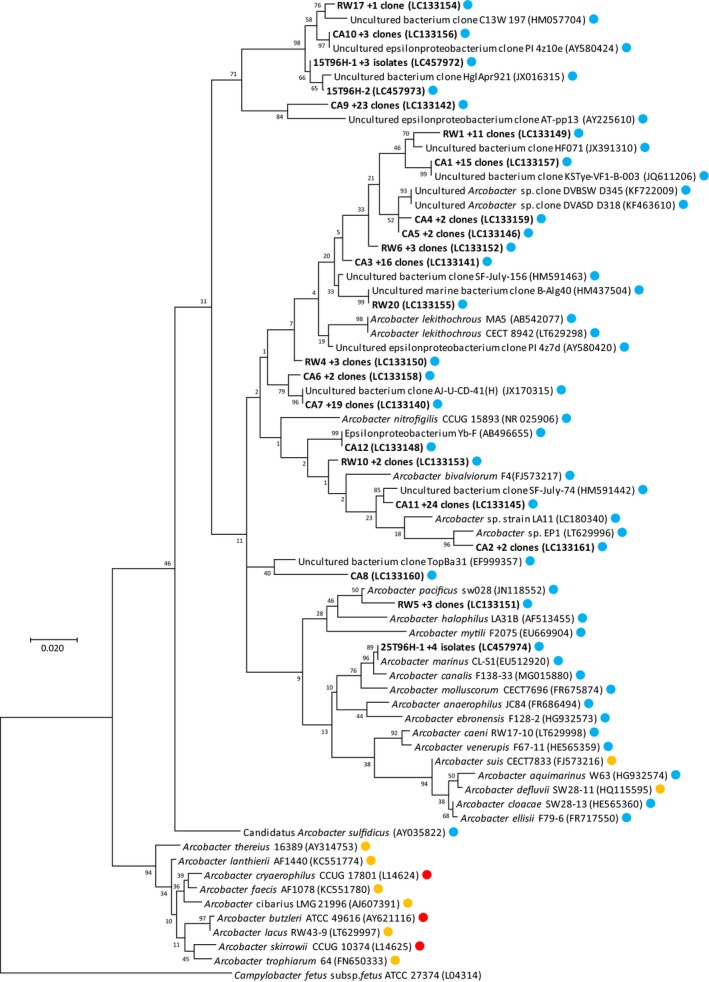
16S rRNA gene‐based phylogenetic tree of *Arcobacter* spp. from abalone and environmental samples. Circles colors indicate origins or pathogenicity of *Arcobacter* spp. as follows: blue, marine habitats, orange, terrestrial environments and red, pathogenic species. The tree was generated using the maximum likelihood (ML) method with 1,000 replicates in the bootstrap analysis. The distances were estimated with the Jukes‐Cantor correction. The tree was rooted with *Campylobacter fetus* subsp. *fetus* ATCC 27374, and gene sequences are followed by GenBank accession numbers in parentheses. Scale bar represents 2% sequence divergence

To prevent bias resulting from culture‐dependent methods in this study, we used *Arcobacter*‐specific clone libraries to directly identify 16S rRNA gene sequences. Twelve OTUs relating to *Arcobacter* were detected from abalones using *Arcobacter*‐specific clone libraries (Table 2), all clustered with previously reported *Arcobacter* sequences (Figure 2). All the OTUs showed similarity to 16S rRNA genes detected from marine environments such as marine invertebrates or seawater, but not from those identified from terrestrial sources such as poultry. Furthermore, these sequences are not classified with any other known *Arcobacter* species. Interestingly, using our analytical approach, the samples from abalones also did not show the presence of pathogenic *Arcobacter* spp., *A. butzleri*, *A. skirrowii,* and *A. cryaerophilus,* commonly isolated or detected from bivalves. Bivalves such as mussels and clams are filter feeders that feed plankton using gills, while abalones feed on brown algae. Thus, they have a more developed digestive system compared to bivalves. The result suggests that gastropods such as abalone may not be host or harbor pathogenic *Arcobacter* species, perhaps due to their different feeding habits and digestive system.

In this study, we employed the ARC94F *Arcobacter*‐specific probe for FISH against cells isolated from abalone for detection and quantification. This ARC94F probe has been used for specific counting of genus *Arcobacter* in seawater (Fera et al., 2008, 2010; Moreno et al., 2003). The ratio of ARC94F‐positive cells suggested that *Arcobacter* might be a common bacterial genus in abalones (6.96%, Table 3). *Haliotis gigantea* appears to be a habitat for *Arcobacter* species, but the role and effect on their hosts are still unclear.

Regarding the cultivation of *Arcobacter* spp., several conditions have been introduced, such as altering the NaCl concentration (Salas‐Massó et al., 2016) or the requirement of sea salts (Diéguez, Balboa, Magnesen, & Romalde, 2017). Hence, we used artificial sea salt instead of NaCl and added more than 2.5% sea salt during isolation. In addition, the incubation temperature used for *Arcobacter* isolation was set to 15 or 25°C, which are closer to the seawater temperatures of the natural habitat of the abalones at Ise Bay, Mie prefecture, against common methods (30 to 37°C: Vandamme et al., 1991; Houf et al., 2000; Collado, Guarro, et al., 2009; Merga et al., 2011; Salas‐Massó et al., 2016; Salas‐Massó, Figueras, Andree, & Furones, 2018; Laishram et al., 2016; González et al., 2017). As a result, 10 *Arcobacter* isolates (five known and five novels) were recovered from samples 15T96H and 25T96H. These were classified into three genotypes based on 97% sequence similarity (15T96H‐1, 15T96H‐2, and 25T96H‐1). The 16S rRNA gene of 25T96H‐1 had a high similarity of 99.2%–100% to *A. marinas*, which has been isolated from a mixture of seawater and starfish (Kim, Hwang, & Cho, 2010). In contrast, the isolates 15T96H‐1 and 15T96H‐2 had no closely related sequences in all other known *Arcobacter* isolates. Including comparisons with uncultured clones, the 16S rRNA genes of 15T96H‐1 or 1596H‐2 have related to an uncultured clone detected from the Pacific oyster *Crassostrea gigas*, an invertebrate living in shallow water (Madigan et al., 2014) and marine bulk water (Teeling et al., 2012). Both 15T96H‐1 and 15T96H‐2 were isolated only at the 15°C incubation temperature. From these observations, we believe that 15T96H‐1 and 15T96H‐2 will be able to be isolated from various marine invertebrates with sea salt medium at lower temperatures. In terms of incubation time, no *Arcobacter* species were isolated within 2 days of incubation. This implies that when incubation temperature is lower than 37°C, *Arcobacter* requires more than 96 hr to grow before colonies can be detected. There are still many species of *Arcobacter* detectable by molecular methods in abalones that are not cultivable. We feel that isolation methods should be improved to obtain these uncultured *Arcobacter* species.

In conclusion, we succeeded in detecting several new *Arcobacter* genotypes from abalone using *Arcobacter*‐specific 16S rRNA gene libraries. Furthermore, since most of the clones showed low similarity with other known *Arcobacter* spp. and no pathogenic *Arcobacter* were detected or isolated from abalone, we need further investigations for uncultured *Arcobacter* spp. which remains to be determined in *H. gigantea*.

## CONFLICT OF INTERESTS

All authors declare no conflict of interest.

## AUTHOR CONTRIBUTIONS

YM, SI, and RO performed the experiments; RT, TM, and SF designed and supervised the study; YM wrote the paper. All authors read and approved the final manuscript.

## ETHICS STATEMENT

None required.

## Data Availability

Raw sequencing data are available at the NCBI website (http://www.ncbi.nlm.nih.gov/). Accession numbers of 16S rRNA gene sequence data from *Arcobacter‐*specific clone libraries are LC133140–LC133161, and from bacteria isolations, LC457972–LC457974.

## References

[mbo3890-bib-0001] Amann, R. I. , Binder, B. J. , Olson, R. J. , Chisholm, S. W. , Devereux, R. , & Stahl, D. A. (1990). Combination of 16S rRNA‐targeted oligonucleotide probes with flow cytometry for analyzing mixed microbial populations. Applied and Environment Microbiology, 56, 1919–1925. 10.1128/aem.56.6.1919-1925.1990 PMC184531 2200342

[mbo3890-bib-0002] Atabay, H. I. , & Corry, J. E. (1997). The prevalence of campylobacters and arcobacters in broiler chickens. Journal of Applied Microbiology, 83, 619–626. 10.1046/j.1365-2672.1997.00277.x 9418023

[mbo3890-bib-0003] Brightwell, G. , Mowat, E. , Clemens, R. , Boerema, J. , Pulford, D. J. , & On, S. L. (2007). Development of a multiplex and real time PCR assay for the specific detection of *Arcobacter butzleri* and *Arcobacter* *cryaerophilus* . Journal of Microbiol Methods, 68, 318–325. 10.1016/j.mimet.2006.09.008 17055091

[mbo3890-bib-0004] Collado, L. , Cleenwerck, I. , Van Trappen, S. , De Vos, P. , & Figueras, M. J. (2009). *Arcobacter mytili* sp. nov., an indoxyl acetate‐hydrolysis‐negative bacterium isolated from mussels. International Journal of Systematic and Evolutionary Microbiology, 59, 1391–1396. 10.1099/ijs.0.003749-0 19502322

[mbo3890-bib-0005] Collado, L. , & Figueras, M. J. (2011). Taxonomy, epidemiology, and clinical relevance of the genus *Arcobacter* . Clinical Microbiology Reviews, 24, 174–192. 10.1128/cmr.00034-10 21233511 PMC3021208

[mbo3890-bib-0006] Collado, L. , Guarro, J. , & Figueras, M. J. (2009). Prevalence of *Arcobacter* in meat and shellfish. Journal of Food Protection, 72, 1102–1106. 10.4315/0362-028X-72.5.1102 19517742

[mbo3890-bib-0007] Collado, L. , Levican, A. , Perez, J. , & Figueras, M. J. (2011). *Arcobacter defluvii* sp. nov., isolated from sewage samples. International Journal of Systematic and Evolutionary Microbiology, 61, 2155–2161. 10.1099/ijs.0.025668-0 20889767

[mbo3890-bib-0008] De Smet, S. , Vandamme, P. , De Zutter, L. , On, S. L. , Douidah, L. , & Houf, K. (2011). *Arcobacter trophiarum* sp. nov., isolated from fattening pigs. International Journal of Systematic and Evolutionary Microbiology, 61, 356–361. 10.1099/ijs.0.022665-0 20305065

[mbo3890-bib-0009] Diéguez, A. L. , Balboa, S. , Magnesen, T. , & Romalde, J. L. (2017). *Arcobacter lekithochrous* sp. nov., isolated from a molluscan hatchery. International Journal of Systematic and Evolutionary Microbiology, 67, 1327–1332. 10.1099/ijsem.0.001809 28109200

[mbo3890-bib-0010] Donachie, S. P. , Bowman, J. P. , On, S. L. , & Alam, M. (2005). *Arcobacter halophilus* sp. nov., the first obligate halophile in the genus *Arcobacter* . International Journal of Systematic and Evolutionary Microbiology, 55, 1271–1277. 10.1099/ijs.0.63581-0 15879267

[mbo3890-bib-0011] Fera, M. T. , Gugliandolo, C. , Lentini, V. , Favaloro, A. , Bonanno, D. , La Camera, E. , & Maugeri, T. L. (2010). Specific detection of *Arcobacter* spp. in estuarine waters of Southern Italy by PCR and fluorescent in situ hybridization. Letters in Applied Microbiology, 50, 65–70. 10.1111/j.1472-765x.2009.02767.x 19929906

[mbo3890-bib-0012] Fera, M. T. , Maugeri, T. L. , Gugliandolo, C. , La Camera, E. , Lentini, V. , Favaloro, A. , … Carbone, M. (2008). Induction and resuscitation of viable nonculturable *Arcobacter butzleri* cells. Applied and Environment Microbiology, 74, 3266–3268. 10.1128/aem.00059-08 PMC2394943 18378639

[mbo3890-bib-0013] Figueras, M. J. , Collado, L. , Levican, A. , Perez, J. , Solsona, M. J. , & Yustes, C. (2011). *Arcobacter molluscorum* sp. nov., a new species isolated from shellfish. Systematic and Applied Microbiology, 34, 105–109. 10.1016/j.syapm.2010.10.001 21185143

[mbo3890-bib-0014] Figueras, M. J. , Levican, A. , Collado, L. , Inza, M. I. , & Yustes, C. (2011). *Arcobacter ellisii* sp. nov., isolated from mussels. Systematic and Applied Microbiology, 34, 414–418. 10.1016/j.syapm.2011.04.004 21723060

[mbo3890-bib-0015] González, A. , Bayas Morejón, I. F. , & Ferrús, M. A. (2017). Isolation, molecular identification and quinolone‐susceptibility testing of *Arcobacter* spp. isolated from fresh vegetables in Spain. Food Microbiology, 65, 279–283. 10.1016/j.fm.2017.02.011 28400014

[mbo3890-bib-0016] Harmon, K. M. , & Wesley, I. V. (1996). Identification of *Arcobacter* isolates by PCR. Letters in Applied Microbiology, 23, 241–244. 10.1111/j.1472-765x.1996.tb00074.x 8987697

[mbo3890-bib-0017] Ho, H. T. , Lipman, L. J. , & Gaastra, W. (2006). *Arcobacter*, what is known and unknown about a potential foodborne zoonotic agent! Veterinary Microbiology, 115, 1–13. 10.1016/j.vetmic.2006.03.004 16621345

[mbo3890-bib-0018] Houf, K. , De Zutter, L. , Van Hoof, J. , & Vandamme, P. (2002). Assessment of the genetic diversity among *Arcobacters* isolated from poultry products by using two PCR‐based typing methods. Applied and Environment Microbiology, 68, 2172–2178. 10.1128/aem.68.5.2172-2178.2002 PMC127564 11976086

[mbo3890-bib-0019] Houf, K. , On, S. L. , Coenye, T. , Debruyne, L. , De Smet, S. , & Vandamme, P. (2009). *Arcobacter thereius* sp. nov., isolated from pigs and ducks. International Journal of Systematic and Evolutionary Microbiology, 59, 2599–2604. 10.1099/ijs.0.006650-0 19622651

[mbo3890-bib-0020] Houf, K. , On, S. L. , Coenye, T. , Mast, J. , Van Hoof, J. , & Vandamme, P. (2005). *Arcobacter cibarius* sp. nov., isolated from broiler carcasses. International Journal of Systematic and Evolutionary Microbiology, 55, 713–717. 10.1099/ijs.0.63103-0 15774649

[mbo3890-bib-0021] Houf, K. , Tutenel, A. , De Zutter, L. , Van Hoof, J. , & Vandamme, P. (2000). Development of a multiplex PCR assay for the simultaneous detection and identification of *Arcobacter butzleri*, *Arcobacter cryaerophilus* and *Arcobacter skirrowii* . FEMS Microbiology Letters, 193, 89–94. 10.1016/s0378-1097(00)00461-4 11094284

[mbo3890-bib-0022] Huber, T. , Faulkner, G. , & Hugenholtz, P. (2004). Bellerophon: A program to detect chimeric sequences in multiple sequence alignments. Bioinformatics, 20, 2317–2319. 10.1093/bioinformatics/bth226 15073015

[mbo3890-bib-0023] Hume, M. E. , Harvey, R. B. , Stanker, L. H. , Droleskey, R. E. , Poole, T. L. , & Zhang, H. B. (2001). Genotypic variation among *Arcobacter* isolates from a farrow‐to‐finish swine facility. Journal of Food Protection, 64, 645–651. 10.4315/0362-028X-64.5.645 11347994

[mbo3890-bib-0024] Kepner, R. L. Jr. , & Pratt, J. R. (1994). Use of fluorochromes for direct enumeration of total bacteria in environmental samples: Past and present. Microbiological Reviews, 58, 603–615. 7854248 10.1128/mr.58.4.603-615.1994 PMC372983

[mbo3890-bib-0025] Khan, I. U. H. , Cloutier, M. , Libby, M. , Lapen, D. R. , Wilkes, G. , & Topp, E. (2017). Enhanced single‐tube multiplex PCR assay for detection and identification of six *Arcobacter* species. Journal of Applied Microbiology, 123, 1522–1532. 10.1111/jam.13597 28960631

[mbo3890-bib-0026] Kiehlbauch, J. A. , Brenner, D. J. , Nicholson, M. A. , Baker, C. N. , Patton, C. M. , Steigerwalt, A. G. , & Wachsmuth, I. K. (1991). *Campylobacter butzleri* sp. nov. isolated from humans and animals with diarrheal illness. Journal of Clinical Microbiology, 29, 376–385. 2007646 10.1128/jcm.29.2.376-385.1991 PMC269771

[mbo3890-bib-0027] Kim, H. M. , Hwang, C. Y. , & Cho, B. C. (2010). *Arcobacter marinus* sp. nov. International Journal of Systematic and Evolutionary Microbiology, 60, 531–536. 10.1099/ijs.0.007740-0 19654359

[mbo3890-bib-0028] Kimura, M. (1980). A simple method for estimating evolutionary rates of base substitutions through comparative studies of nucleotide sequences. Journal of Molecular Evolution, 16, 111–120. 10.1007/bf01731581 7463489

[mbo3890-bib-0029] Kumar, S. , Stecher, G. , & Tamura, K. (2016). MEGA7: Molecular evolutionary genetics analysis version 7.0 for bigger datasets. Molecular Biology and Evolution, 33, 1870–1874. 10.1093/molbev/msw054 27004904 PMC8210823

[mbo3890-bib-0030] Laishram, M. , Rathlavath, S. , Lekshmi, M. , Kumar, S. , & Nayak, B. B. (2016). Isolation and characterization of *Arcobacter* spp. from fresh seafood and the aquatic environment. International Journal of Food Microbiology, 232, 87–89. 10.1016/j.ijfoodmicro.2016.05.018 27261768

[mbo3890-bib-0031] Levican, A. , Collado, L. , Aguilar, C. , Yustes, C. , Diéguez, A. L. , Romalde, J. L. , & Figueras, M. J. (2012). *Arcobacter bivalviorum* sp. nov. and *Arcobacter venerupis* sp. nov., new species isolated from shellfish. Systematic and Applied Microbiology, 35, 133–138. 10.1016/j.syapm.2012.01.002 22401779

[mbo3890-bib-0032] Levican, A. , Collado, L. , & Figueras, M. J. (2013). *Arcobacter cloacae* sp. nov. and *Arcobacter suis* sp. nov., two new species isolated from food and sewage. Systematic and Applied Microbiology, 36, 22–27. 10.1016/j.syapm.2012.11.003 23265195

[mbo3890-bib-0033] Levican, A. , Collado, L. , Yustes, C. , Aguilar, C. , & Figueras, M. J. (2014). Higher water temperature and incubation under aerobic and microaerobic conditions increase the recovery and diversity of *Arcobacter* spp. from shellfish. Applied and Environment Microbiology, 80, 385–391. 10.1128/AEM.03014-13 PMC3911005 24185851

[mbo3890-bib-0034] Levican, A. , Rubio‐Arcos, S. , Martinez‐Murcia, A. , Collado, L. , & Figueras, M. J. (2015). *Arcobacter ebronensis* sp. nov. and *Arcobacter aquimarinus* sp. nov., two new species isolated from marine environment. Systematic and Applied Microbiology, 38, 30–35. 10.1016/j.syapm.2014.10.011 25497285

[mbo3890-bib-0035] Madigan, T. L. , Bott, N. J. , Torok, V. A. , Percy, N. J. , Carragher, J. F. , de Barros Lopes, M. A. , & Kiermeier, A. (2014). A microbial spoilage profile of half shell Pacific oysters (*Crassostrea gigas*) and Sydney rock oysters (*Saccostrea glomerata*). Food Microbiology, 38, 219–227. 10.1016/j.fm.2013.09.005 24290646

[mbo3890-bib-0036] McClung, C. R. , Patriquin, D. G. , & Davis, R. E. (1983). *Campylobacter nitrofigilis* sp. nov., a nitrogen‐fixing bacterium associated with roots of *Spartina alterniflora Loisel* . International Journal of Systematic Bacteriology, 33, 605–612. 10.1099/00207713-33-3-605

[mbo3890-bib-0037] Merga, J. Y. , Leatherbarrow, A. J. , Winstanley, C. , Bennett, M. , Hart, C. A. , Miller, W. G. , & Williams, N. J. (2011). Comparison of *Arcobacter* isolation methods, and diversity of *Arcobacter* spp. in Cheshire, United Kingdom. Applied and Environmental Microbiology, 77, 1646–1650. 10.1128/AEM.01964-10 21193675 PMC3067278

[mbo3890-bib-0038] Meziti, A. , Mente, E. , & Kormas, K. A. (2012). Gut bacteria associated with different diets in reared *Nephrops norvegicus* . Systematic and Applied Microbiology, 35, 473–482. 10.1016/j.syapm.2012.07.004 23040460

[mbo3890-bib-0039] Moreno, Y. , Botella, S. , Alonso, J. L. , Ferrús, M. A. , Hernández, M. , & Hernández, J. (2003). Specific detection of *Arcobacter* and *Campylobacter* strains in water and sewage by PCR and fluorescent in situ hybridization. Applied and Environment Microbiology, 69, 1181–1186. 10.1128/aem.69.2.1181-1186.2003 PMC143587 12571045

[mbo3890-bib-0040] Mottola, A. , Bonerba, E. , Figueras, M. J. , Pérez‐Cataluña, A. , Marchetti, P. , Serraino, A. , … Di Pinto, A. (2016). Occurrence of potentially pathogenic arcobacters in shellfish. Food Microbiology, 57, 23–27. 10.1016/j.fm.2015.12.010 27052698

[mbo3890-bib-0041] Neill, S. D. , Campbell, J. N. , O'Brien, J. J. , Weatherup, S. T. C. , & Ellis, W. A. (1985). Taxonomic position of *Campylobacter cryaerophila* sp. nov. International Journal of Systematic Bacteriology, 35, 342–356. 10.1099/00207713-35-3-342

[mbo3890-bib-0042] On, S. L. , Atabay, H. I. , Amisu, K. O. , Coker, A. O. , & Harrington, C. S. (2004). Genotyping and genetic diversity of *Arcobacter butzleri* by amplified fragment length polymorphism (AFLP) analysis. Letters in Applied Microbiology, 39, 347–352. 10.1111/j.1472-765x.2004.01584.x 15355537

[mbo3890-bib-0043] On, S. L. , Harrington, C. S. , & Atabay, H. I. (2003). Differentiation of *Arcobacter* species by numerical analysis of AFLP profiles and description of a novel *Arcobacter* from pig abortions and turkey faeces. Journal of Applied Microbiology, 95, 1096–1105. 10.1046/j.1365-2672.2003.02100.x 14633039

[mbo3890-bib-0044] Ootsubo, M. , Shimizu, T. , Tanaka, R. , Sawabe, T. , Tajima, K. , & Ezura, Y. (2003). Seven‐hour fluorescence in situ hybridization technique for enumeration of Enterobacteriaceae in food and environmental water sample. Journal of Applied Microbiology, 95, 1182–1190. 10.1046/j.1365-2672.2003.02051.x 14632990

[mbo3890-bib-0045] Park, S. , Jung, Y. T. , Kim, S. , & Yoon, J. H. (2016). *Arcobacter acticola* sp. nov., isolated from seawater on the East Sea in South Korea. Journal of Microbiology, 54, 655–659. 10.1007/s12275-016-6268-4 27687227

[mbo3890-bib-0046] Perez‐Cataluña, A. , Salas‐Masso, N. , & Figueras, M. J. (2018a). *Arcobacter canalis* sp. nov., isolated from a water canal contaminated with urban sewage. International Journal of Systematic and Evolutionary Microbiology, 68, 1258–1264. 10.1099/ijsem.0.002662 29488868

[mbo3890-bib-0047] Pérez‐Cataluña, A. , Salas‐Massó, N. , & Figueras, M. J. (2018b). *Arcobacter lacus* sp. nov. and *Arcobacter caeni* sp. nov., two novel species isolated from reclaimed water. Systematic and Applied Microbiology. 10.1099/ijsem.0.003101 30394871

[mbo3890-bib-0048] Rathlavath, S. , Kohli, V. , Singh, A. S. , Lekshmi, M. , Tripathi, G. , Kumar, S. , & Nayak, B. B. (2017). Virulence genotypes and antimicrobial susceptibility patterns of *Arcobacter butzleri* isolated from seafood and its environment. International Journal of Food Microbiology, 263, 32–37. 10.1016/j.ijfoodmicro.2017.10.005 29028568

[mbo3890-bib-0049] Rathlavath, S. , Kumar, S. , & Nayak, B. B. (2017). Comparative isolation and genetic diversity of *Arcobacter* sp. from fish and the coastal environment. Letters in Applied Microbiology, 65, 42–49. 10.1111/lam.12743 28394467

[mbo3890-bib-0050] Roller, C. , Wagner, M. , Amann, R. , Ludwig, W. , & Schleifer, K. H. (1994). In situ probing of Gram‐positive bacteria with high DNA G + C content using 23S rRNA‐targeted oligonucleotides. Microbiology, 140, 2849–2858. 10.1099/00221287-140-10-2849 8000548

[mbo3890-bib-0051] Romero, J. , García‐Varela, M. , Laclette, J. P. , & Espejo, R. T. (2002). Bacterial 16S rRNA gene analysis revealed that bacteria related to *Arcobacter* spp. constitute an abundant and common component of the oyster microbiota (*Tiostrea chilensis*). Microbial Ecology, 44, 365–371. 10.1007/s00248-002-1063-7 12399898

[mbo3890-bib-0052] Salas‐Massó, N. , Andree, K. B. , Furones, M. D. , & Figueras, M. J. (2016). Enhanced recovery of *Arcobacter* spp. using NaCl in culture media and re‐assessment of the traits of *Arcobacter marinus* and *Arcobacter halophilus* isolated from marine water and shellfish. Science of the Total Environment, 566–567, 1355–1361. 10.1016/j.scitotenv.2016.05.197 27282494

[mbo3890-bib-0053] Salas‐Massó, N. , Figueras, M. J. , Andree, K. B. , & Furones, M. D. (2018). Do the *Escherichia coli* European Union shellfish safety standards predict the presence of *Arcobacter* spp., a potential zoonotic pathogen? Science of the Total Environment, 624, 1171–1179. 10.1016/j.scitotenv.2017.12.178 29929229

[mbo3890-bib-0054] Sasi Jyothsna, T. S. , Rahul, K. , Ramaprasad, E. V. , Sasikala, C. H. , & Ramana, C. V. (2013). *Arcobacter anaerophilus* sp. nov., isolated from an estuarine sediment and emended description of the genus *Arcobacter* . International Journal of Systematic and Evolutionary Microbiology, 63, 4619–4625. 10.1099/ijs.0.054155-0 23918794

[mbo3890-bib-0055] Snaidr, J. , Amann, R. , Huber, I. , Ludwig, W. , & Schleifer, K. H. (1997). Phylogenetic analysis and in situ identification of bacteria in activated sludge. Applied and Environment Microbiology, 63, 2884–2896. 10.1128/aem.63.7.2884-2896.1997 PMC168584 9212435

[mbo3890-bib-0056] Stackebrandt, E. , & Goebel, B. M. (1994). Taxonomic note: A place for DNA‐DNA reassociation and 16S rRNA sequence analysis in the present species definition in bacteriology. International Journal of Systematic Bacteriology, 44, 846–849. 10.1099/00207713-44-4-846

[mbo3890-bib-0057] Tanaka, R. , Cleenwerck, I. , Mizutani, Y. , Iehata, S. , Bossier, P. , & Vandamme, P. (2017). *Arcobacter haliotis* sp. nov., isolated from abalone species *Haliotis gigantea* . International Journal of Systematic and Evolutionary Microbiology, 67, 3050–3056. 10.1099/ijsem.0.002080 28820118

[mbo3890-bib-0058] Tanaka, R. , Ootsubo, M. , Sawabe, T. , Ezura, Y. , & Tajima, K. (2004). Biodiversity and in situ abundance of gut microflora of abalone (*Haliotis discus hannai*) determined by culture‐independent techniques. Aquaculture, 241, 453–463. 10.1016/j.aquaculture.2004.08.032

[mbo3890-bib-0059] Teeling, H. , Fuchs, B. M. , Becher, D. , Klockow, C. , Gardebrecht, A. , Bennke, C. M. , … Amann, R. (2012). Substrate‐controlled succession of marine bacterioplankton populations induced by a phytoplankton bloom. Science, 336, 608–611. 10.1126/science.1218344 22556258

[mbo3890-bib-0060] Thompson, J. D. , Higgins, D. G. , & Gibson, T. J. (1994). CLUSTAL W: Improving the sensitivity of progressive multiple sequence alignment through sequence weighting, position‐specific gap penalties and weight matrix choice. Nucleic Acids Research, 22, 4673–4680. 10.1093/nar/22.22.4673 7984417 PMC308517

[mbo3890-bib-0061] Vandamme, P. , Falsen, E. , Rossau, R. , Hoste, B. , Segers, P. , Tytgat, R. , & De Ley, J. (1991). Revision of *Campylobacter*, *Helicobacter*, and *Wolinella* taxonomy: Emendation of generic descriptions and proposal of *Arcobacter* gen. nov. International Journal of Systematic Bacteriology, 41, 88–103. 10.1099/00207713-41-1-88 1704793

[mbo3890-bib-0062] Vandamme, P. , Giesendorf, B. A. , van Belkum, A. , Pierard, D. , Lauwers, S. , Kersters, K. , … Quint, W. G. (1993). Discrimination of epidemic and sporadic isolates of *Arcobacter butzleri* by polymerase chainreaction‐mediated DNA fingerprinting. Journal of Clinical Microbiology, 31, 3317–3319. 8308127 10.1128/jcm.31.12.3317-3319.1993 PMC266416

[mbo3890-bib-0063] Vandamme, P. , Vancanneyt, M. , Pot, B. , Mels, L. , Hoste, B. , Dewettinck, D. , … Goossens, H. (1992). Polyphasic taxonomic study of the emended genus *Arcobacter* with *Arcobacter butzleri* comb. nov. and *Arcobacter skirrowii* sp. nov., an aerotolerant bacterium isolated from veterinary specimens. International Journal of Systematic Bacteriology, 42, 344–356. 10.1099/00207713-42-3-344 1503968

[mbo3890-bib-0064] Vicente‐Martins, S. , Oleastro, M. , Domingues, F. C. , & Ferreira, S. (2018). *Arcobacter* spp. at retail food from Portugal: Prevalence, genotyping and antibiotics resistance. Food Control, 85, 107–112. 10.1016/j.foodcont.2017.09.024

[mbo3890-bib-0065] Whiteduck‐Léveillée, K. , Whiteduck‐Léveillée, J. , Cloutier, M. , Tambong, J. T. , Xu, R. , Topp, E. , … Khan, I. U. (2015). *Arcobacter lanthieri* sp. nov., isolated from pig and dairy cattle manure. International Journal of Systematic and Evolutionary Microbiology, 65, 2709–2716. 10.1099/ijs.0.000318 25977280

[mbo3890-bib-0066] Whiteduck‐Léveillée, K. , Whiteduck‐Léveillée, J. , Cloutier, M. , Tambong, J. T. , Xu, R. , Topp, E. , … Khan, I. U. (2016). Identification, characterization and description of *Arcobacter faecis* sp. nov., isolated from a human waste septic tank. Systematic and Applied Microbiology, 39, 93–99. 10.1016/j.syapm.2015.12.002 26723853

[mbo3890-bib-0067] Zhang, Z. , Yu, C. , Wang, X. , Yu, S. , & Zhang, X. H. (2016). *Arcobacter pacificus* sp. nov., isolated from seawater of the South Pacific Gyre. International Journal of Systematic and Evolutionary Microbiology, 66, 542–547. 10.1099/ijsem.0.000751 26556763

